# Clustering of the K^+^ channel GORK of Arabidopsis parallels its gating by extracellular K^+^

**DOI:** 10.1111/tpj.12471

**Published:** 2014-04-02

**Authors:** Cornelia Eisenach, Maria Papanatsiou, Ellin-Kristina Hillert, Michael R Blatt

**Affiliations:** Laboratory of Plant Physiology and Biophysics, Institute of Molecular, Cell and Systems Biology, University of GlasgowBower Building, Glasgow, G12 8QQ, UK

**Keywords:** GORK K^+^ channel – outward-rectifying, membrane vesicle traffic, plasma membrane, confocal microscopy, K^+^ concentration – extracellular, channel gating – K^+^-dependent, Arabidopsis

## Abstract

GORK is the only outward-rectifying Kv-like K^+^ channel expressed in guard cells. Its activity is tightly regulated to facilitate K^+^ efflux for stomatal closure and is elevated in ABA in parallel with suppression of the activity of the inward-rectifying K^+^ channel KAT1. Whereas the population of KAT1 is subject to regulated traffic to and from the plasma membrane, nothing is known about GORK, its distribution and traffic *in vivo*. We have used transformations with fluorescently-tagged GORK to explore its characteristics in tobacco epidermis and Arabidopsis guard cells. These studies showed that GORK assembles in puncta that reversibly dissociated as a function of the external K^+^ concentration. Puncta dissociation parallelled the gating dependence of GORK, the speed of response consistent with the rapidity of channel gating response to changes in the external ionic conditions. Dissociation was also suppressed by the K^+^ channel blocker Ba^2+^. By contrast, confocal and protein biochemical analysis failed to uncover substantial exo- and endocytotic traffic of the channel. Gating of GORK is displaced to more positive voltages with external K^+^, a characteristic that ensures the channel facilitates only K^+^ efflux regardless of the external cation concentration. GORK conductance is also enhanced by external K^+^ above 1 mm. We suggest that GORK clustering in puncta is related to its gating and conductance, and reflects associated conformational changes and (de)stabilisation of the channel protein, possibly as a platform for transmission and coordination of channel gating in response to external K^+^.

## Introduction

Control of membrane transport is central to solute flux for stomatal regulation. Membrane transport of the predominant, osmotically-active ion, K^+^, is of particular importance and is mediated largely by voltage-dependent K^+^ channels. Inward-rectifying K^+^ channels mediate K^+^ uptake during stomatal opening, while outward-rectifying K^+^ channels mediate its loss during stomatal closing (Blatt and Thiel, 1993; Blatt, 2000; Lebaudy *et al*., 2007; Dreyer and Blatt, 2009). In Arabidopsis, the former are dominated by the inward-rectifying K^+^ channel KAT1, which is highly-expressed in guard cells, and to a lesser extent the related channel KAT2. K^+^ efflux across the plasma membrane is facilitated by GORK, which is the only outward rectifying K^+^ -channel known to be expressed in the guard cells (Ache *et al*., 2000; Hosy *et al*., 2003; Dreyer and Blatt, 2009).

KAT1 and GORK belong to the Kv superfamily of voltage-gated ion channels that are found also in archaea, insects and mammals. These channels open and close, a process referred to as ‘gating’, in response to membrane voltage. Outward-rectifying K^+^ channels of plants, including GORK, also gate in response to the extracellular K^+^ concentration. Increasing the extracellular K^+^ concentration displaces the voltage-dependence of gating to more positive voltages in parallel with the equilibrium voltage for K^+^ (*E*_K_) and, at concentrations above 1 mm, increases the maximum ensemble conductance (Blatt, 1988; Blatt and Gradmann, 1997; Ache *et al*., 2000; Johansson *et al*., 2006). Potassium is the most abundant ionic species in plants; its concentration outside, and its equilibrium, often dominates the membrane voltage in plants. Gating by extracellular K^+^ therefore ensures that these channels activate at a voltage relative to *E*_K_ and adjusts K^+^ efflux through the channels in a compensatory manner across a wide range of K^+^ concentrations (Blatt, 1988; Blatt and Gradmann, 1997).

The activities of guard cell K^+^ channels are subject to a number of regulatory stimuli, including hormones such as abscisic acid (ABA) (Blatt, 2000; Blatt *et al*., 2007; Kim *et al*., 2010; Roelfsema and Hedrich, 2010), auxin (Thiel *et al*., 1993; Blatt and Thiel, 1994; Bauly *et al*., 2000), metabolites (Hedrich *et al*., 2001; Wang and Blatt, 2011), and pathogen elicitors (Blatt *et al*., 1999). Many of these stimuli act through intermediates of cytosolic-free [Ca^2+^] ([Ca^2+^]_i_), pH and protein (de-)phosphorylation (Blatt *et al*., 1990; Blatt and Grabov, 1999; Blatt, 2000; Kim *et al*., 2010). Channel responses to these intermediates are usually very rapid and are underpinned by changes in channel gating, but over longer time periods can extend to membrane vesicle traffic and changes in channel population at the membrane. Much less is known of the scope of channel regulation by vesicle traffic or its regulation. To date, only the KAT1 K^+^ channel has been studied in any detail (Hurst *et al*., 2004; Meckel *et al*., 2005; Sutter *et al*., 2006, 2007; Eisenach *et al*., 2012). KAT1 is localised at the plasma membrane in punctate domains from which ABA and [Ca^2+^]_i_ trigger its endocytosis. Its recycling to the plasma membrane depends on the vesicle-trafficking protein SYP121 (=SYR1/PEN1) (Leyman *et al*., 1999) and underpins the phenomenon of so-called ‘programmed closure’ of stomata (Eisenach *et al*., 2012). By contrast, nothing is known of the localisation, distribution and trafficking of the outward-rectifying K^+^ channels, including GORK. Here we show that GORK is localised in discrete puncta at the plasma membrane that are similar to, but distinct from, those reported previously for KAT1-GFP. Unexpectedly, GORK-GFP puncta proved to be insensitive to ABA or the presence of SYP121, but channel distribution was affected by increasing external K^+^ concentrations in parallel with the dependence of channel gating on the cation. We propose that the gating of GORK with external K^+^ concentration is associated with a re-organisation of the channel proteins in clusters within the plane of the plasma membrane.

## Results

### GORK-GFP is a functional outward rectifying K^+^ channel that localises in puncta

We generated GORK-GFP constructs for expression under the control of the Ubiquitin-10 gene promoter to give moderate over-expression *in vivo* (Grefen *et al*., 2010b) and verified functionality of the recombinant protein after expression in *Xenopus* oocytes. Oocytes injected with GORK-GFP cRNA showed an outward current (Figure S1) when clamped to voltages positive of *E*_K_, with current relaxations typical of the GORK channel and block by TEA^+^ (Figure S2). Relative conductances (*G*/*G*_max_) were well-fitted to a Boltzmann function (see Figure S1) with a half-maximal activation voltage (*V*_1/2_) that was displaced in parallel with *E*_K_, much as has previously been shown for GORK (Ache *et al*., 2000), the outward-rectifier of *Vicia* (Blatt, 1988; Blatt and Gradmann, 1997) and for SKOR, the close homolog of the GORK K^+^ channel (Johansson *et al*., 2006).

**Figure 1:**
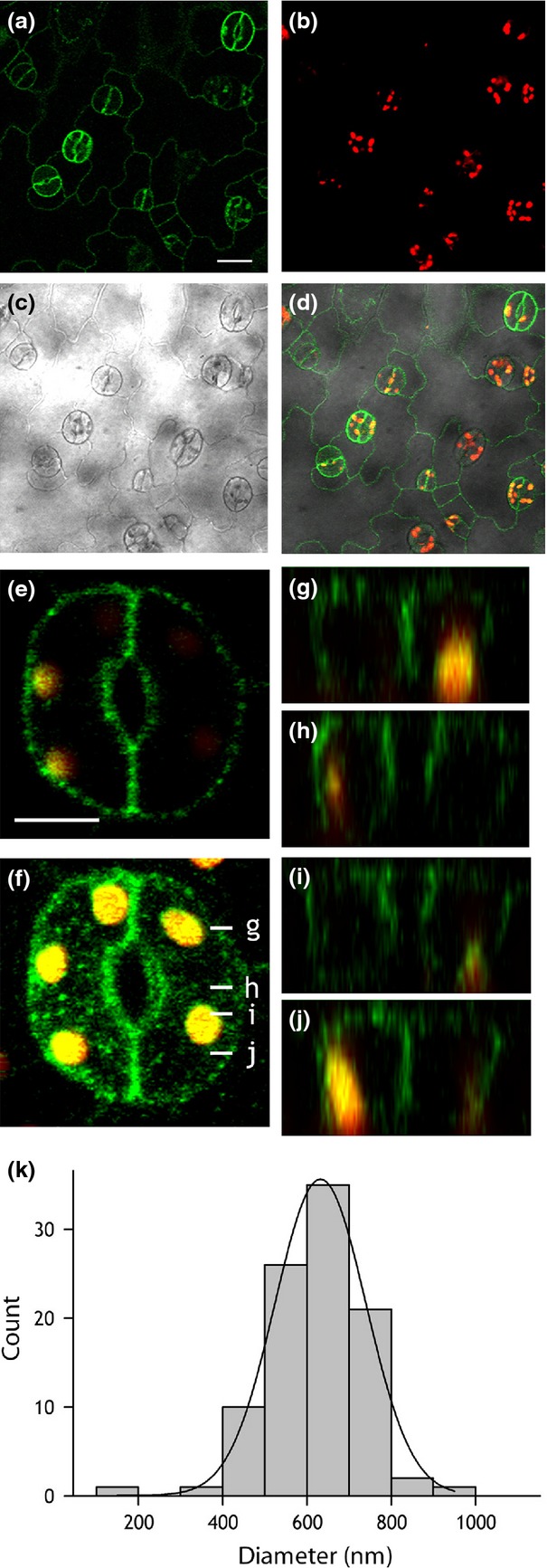
GORK localises in puncta at the periphery of Arabidopsis guard cells and epidermal cells.(a–d) Confocal fluorescence images of GORK-GFP (green, a), chlorophyll fluorescence (red, b), bright field (c) and the corresponding overlay image (d) from the epidermis of an Arabidopsis leaf expressing *pUBQ10::GORK-GFP*. Scale bar, 20 µm.(e) Lateral optical section through a single pair of Arabidopsis guard cells showing punctate distribution of GORK-GFP (green) and chloroplasts (yellow). Scale bar, 5 µm.(f) 3D projection of the same guard cell pair in (e) reconstructed from a *z*-stack collected at intervals of 0.8 µm.(g–j) *Z*-plane sections derived from (f) at the positions indicated by white lines in (f).(k) Histogram analysis of the diameters of GORK-GFP puncta. Data were fitted to a gaussian function (solid line), indicating a normal distribution of diameters about a mean of 632 ± 1 nm.

We generated stable transformants with GORK-GFP in wild-type Arabidopsis and in the *syp121* mutant that lacks the vesicle-trafficking (SNARE) protein SYP121 (=SYR1/PEN1) (Leyman *et al*., 1999) and is known to affect K^+^ channel traffic and function (Honsbein *et al*., 2009; Grefen *et al*., 2010a; Eisenach *et al*., 2012). Leaves of T_2_ and T_3_ lines from three independent transformation events were selected; in every case GORK-GFP fluorescence appeared in puncta around the cell periphery (Figure1), both in wild-type and *syp121* mutant backgrounds. Fluorescence was detected in epidermal cells and guard cells (Figure1a), distinct from chloroplast fluorescence (Figure1b), and was localised to the cell periphery (Figure1c–j). Intracellular GORK-GFP signals were observed in 7% (*n* = 72) of stomata, mostly in very young, developing tissue. Analysis of puncta size yielded diameters of of 0.63 ± 0.01 μm (*n* = 97) that were normally distributed and well-removed from the diffraction limit near 300 nm (Figure1k). These dimensions compare favourably with the mean diameter of 0.5 μm observed for microdomains formed by KAT1-GFP in tobacco (Sutter *et al*., 2006). Although similar in size and distribution, the puncta formed by RFP-tagged GORK did not co-localize (Figures S3 and S4) with puncta formed by the GFP-tagged KAT1 K^+^ channel (Sutter *et al*., 2006, 2007). When expressed in tobacco, fluorescence was occasionally observed within epidermal cells; nonetheless, after plasmolysis GORK-GFP fluorescence was observed in Hechtian strands (Figure S5, *arrows*), indicating that the punctate structures were localised to the plasma membrane.

### GORK-GFP puncta are sensitive to the external KCl concentration

**Figure 2:**
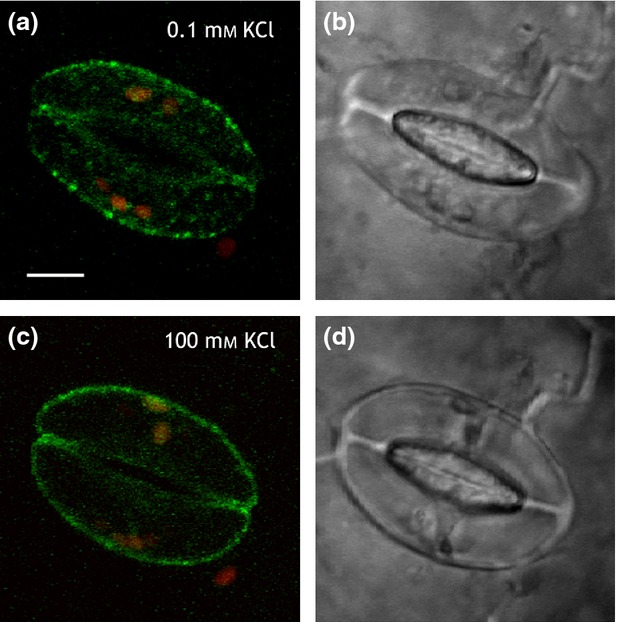
GORK-GFP fluorescence is punctate in 0.1 mmKCl, but is diffuse in 100 mm KCl.GORK-GFP fluorescence (a, c) and brightfield images (b, d) in a pair of guard cells in 0.1 mm KCl (a, b) and after transfer to 100 mm KCl (c, d). Scale bar, 5 µm. Images in (a) and (c) are overlaid, 3D projections of GORK-GFP(green) and chloroplast (red) fluorescence from *z*-stacks taken at intervals of 0.7 µm. Equivalent results were obtained with Hyperpolarising and Depolarising Buffers (see text).

KAT1-GFP is internalised from the plasma membrane following application of ABA (Sutter *et al*., 2007), and by treatments known to elevate [Ca^2+^]_i_ (Grabov and Blatt, 1998), studies showing its recycling by SYP121-mediated traffic underpins programmed stomatal closure (Leyman *et al*., 1999; Eisenach *et al*., 2012). We used the treatments to elevate [Ca^2+^]_i_, anticipating that GORK traffic would complement that of the KAT1 channel. Intact leaves were incubated for 2 h under light after infiltration with Depolarising Buffer (DB) containing 100 mm KCl and 0 CaCl_2_ to open stomata, and stomatal closure was induced by exchange with Hyperpolarising Buffer (HB) containing 0.1 mm KCl and 10 mm CaCl_2_ (Eisenach *et al*., 2012). We found that GORK-GFP did not internalise following HB treatment, unlike KAT1 (Eisenach *et al*., 2012). Instead, the punctate distribution of GORK-GFP was lost in DB, the fluorescence redistributing around the cell periphery. This process was fully reversible on transfer back to HB and was evident in both wild-type and *syp121* mutant Arabidopsis. However, treatments with ABA had no effect on GORK-GFP distribution (Figures S6 and S7). We reasoned that the changes in the punctate distribution observed between DB and HB were likely to be related to the ionic content of the buffer rather than to Ca^2+^ or ABA signalling *per se*. We repeated the experiment with solutions of 100 mm KCl and 0.1 mm KCl alone. Again, we found, GORK-GFP puncta were clearly visible in 0.1 mm KCl (Figure2a,b) but, in the same guard cells, the punctate distribution was lost when the external KCl concentration was raised to 100 mm (Figure2c,d). To exclude an effect of osmotic strength, we repeated these experiments with 0.1 mm KCl including Mannitol to adjust the osmotic strength to that of the 100 mm KCl (200 ± 10 mOsmol). No difference in GORK-GFP distribution was observed between treatments with and without mannitol, thus excluding osmotic effects as the explanation for the changing GORK-GFP distribution.

### GORK-GFP redistribution is not associated with endocytic traffic

**Figure 3:**
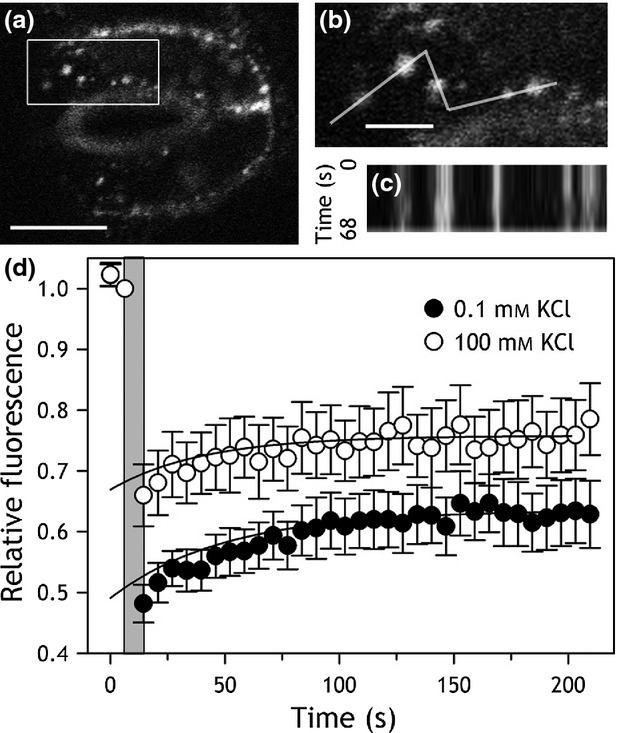
The macroscopic mobility of GORK-GFP at the cell periphery is not affected by extracellular KCl concentration.(a) Tangential section of a guard cell pair with GORK-GFP puncta in 0.1 mm KCl. Scale bar, 5 µm.(b) Close-up of the boxed region in (a) with the linescan for kymographic analysis as indicated. Scale bar, 2 µm.(c) Kymograph over the linescan in (b) collected over a time interval of 68 sec. The *x*-axis corresponds to the linescan dimension and the *y*-axis corresponds to time. The analysis shows that GORK-GFP is positionally stable over this time period.(d) Fluorescence recovery after photobleaching (FRAP) of the GORK-GFP recorded from guard cells in 0.1 mm KCl (closed circles, *n* = 12) and in 100 mm KCl (open circles, *n* = 11). Fluorescence was normalised to the signals at the start of the experiments after correcting for fluorescence decay (Sutter *et al*., 2006, 2007) and fluorescence recoveries after the photobleaching period (grey) were fitted to a single exponential function (solid lines) to derive halftimes and amplitudes for recovery.

The dissolution of KAT1 puncta is marked by a substantial increase in mobility and endocytosis of the channels (Sutter *et al*., 2007; Eisenach *et al*., 2012). To determine whether GORK-GFP dispersal was similarly associated with endocytosis from the plasma membrane, we first examined the mobility of GORK-GFP by time-lapse and by fluorescence recovery after photobleaching (FRAP) analysis. Figure3 illustrates one of eight independent time-lapse experiments with GORK-GFP puncta identified at the stomatal surface in tangential scans (Figure3a,b). Kymographic analysis (Figure3c) yielded no evidence of lateral movement. We used FRAP analysis in Arabidopsis to determine the recovery of fluorescence at the cell periphery, calculating the mobile fraction of GORK-GFP fluorescence from the signal recovery. GORK-GFP was photobleached locally at the periphery of guard cells preincubated in 0.1 and 100 mm KCl, and its recovery within the photobleached regions was monitored. Figure3(d) summarizes the result of 12 independent experiments. FRAP analysis yielded similar results in 0.1 and 100 mm KCl: fluorescence recovered with a mean halftime of 36 ± 4 sec in 0.1 mm KCl and 28 ± 7 sec in 100 mm KCl, a difference that was not significant at the *P* < 0.05 level. Additionally, in both KCl concentrations the GORK-GFP signal was predominantly non-mobile, with a mobile fraction of 9 ± 1% in 100 mm KCl and of 14 ± 6% in 0.1 mm KCl. Again, the difference was not statistically significant at the *P* < 0.05 level.

**Figure 4:**
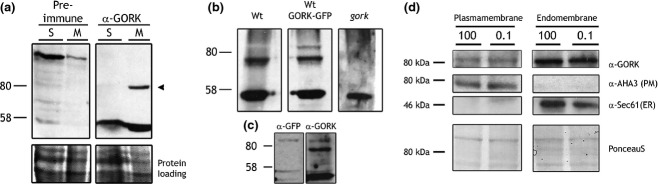
αGORK antibody raised to an N-terminal peptide shows no substantial redistribution of the K^+^ channel between plasma membrane and endomembranes in Arabidopsis.(a) Immunoblot (top) and Ponecau S stain (bottom) of wild-type Arabidopsis soluble (S) and microsomal (M) protein fractions, probed either with pre-immune serum (*left*) and with the purified αGORK antibody (*right*). The αGORK antibody uniquely detected a single band around 80 kDa in the microsomal fraction (*arrowhead, right*).(b) Immunoblot of Arabidopsis microsomal fractions from wild type (Wt), a transgenic line expressing GORK-GFP (GORK-GFP), and the *gork* null mutant. Note the double band in the GORK-GFP fraction corresponds to endogenous GORK and recombinant GORK-GFP. The 80 kDa band is missing from the *gork* mutant. The lower intensity of the GORK-GFP band probably is a consequence of co-suppression of the transgene.(c) Immunoblot of Arabidopsis microsomal fractions from a transgenic line expressing GORK-GFP and probed with αGFP antibody. Note that the antibody detects a specific band above 80 kDa. An additional band is evident on stripping and reprobing with αGORK antibody, consistent with the presence of the native channel.(d) Endogenous GORK is not affected by the external buffer or KCl concentration.Immunoblot and PonceauS stain of plasma membrane and endomembrane protein fractions from aqueous two-phase partitioning of Arabidopsis leaves pretreated with either 0.1 or 100 mm KCl (0.1 and 100, *indicated above*). Molecular weights (*left*) and staining or antibody used for detection (*right*) are indicated. PonceauS stain of the membrane (*bottom*) shows equal protein loading within each gel. The membrane was probed with αGORK antibody (αGORK) before stripping and re-probing, first with an antibody against the plasma membrane H^+^-ATPase AHA3 (αAHA3), and an antibody against the ER-localised translocon Sec61 (αSec61).

We used aqueous, two-phase partitioning as an independent test for GORK redistribution between the plasma membrane and endomembranes, including endocytotic vesicles (Sutter *et al*., 2006, 2007; Honsbein *et al*., 2009). GORK polyclonal antibodies were raised against a synthetic peptide corresponding to a GORK-specific N-terminal amino-acid sequence (Figure S8). The antibodies were purified and verified for specificity in detecting GORK after immunoblotting. Figure4(a) shows an immunoblot of soluble (S) and microsomal (M) protein fractions probed with αGORK antibody and the pre-immune serum. The antibody detected a band near the 80 kDa marker in the microsomal protein fraction and close to the 93.8 kDa predicted for GORK. Analysis of microsomal proteins extracts from wild-type plants, a transgenic GORK-GFP overexpressing line probed with αGFP antibody, and the *gork* mutant confirmed this band as the GORK protein (Figure4b).

We separated plasma membrane and endomembrane fractions after pretreatments of leaf tissues with 0.1 and 100 mm KCl. Figure4(c) shows an immunoblot of plasma membrane and endomembrane protein fractions from one of two independent experiments, each yielding similar results. Equal loading between samples of plasma and of endomembrane fractions was verified by Ponceau staining and purity was confirmed by probing against the plasma membrane marker AHA3 (Pardo and Serrano, 1989) and the endomembrane marker Sec61 (Yuasa *et al*., 2005). GORK bands of comparable intensity were observed in plasma membrane fractions irrespective of pretreatments with 0.1 and 100 mm KCl (Figure4c, *left*). GORK bands were also detected in the endomembrane fractions (Figure4c, *right*). An absolute comparison with the plasma membrane fractions is not possible, because of differences in total protein, but a ratiometric comparison between treatments showed little difference in relative distributions. The band density ratios for GORK between plasma membrane fractions in 0.1 and 100 mm KCl yielded values of 1.3 in the first experiment and 1.1 in the second. Thus, KCl treatments did not have a major effect on GORK distribution to the plasma membrane, indicating that vesicle traffic to and from the plasma membrane was unlikely to contribute appreciably to the changes observed in GORK-GFP distribution *in vivo*.

### GORK redistribution is fast and mirrors alkali cation-dependent channel gating

**Figure 5:**
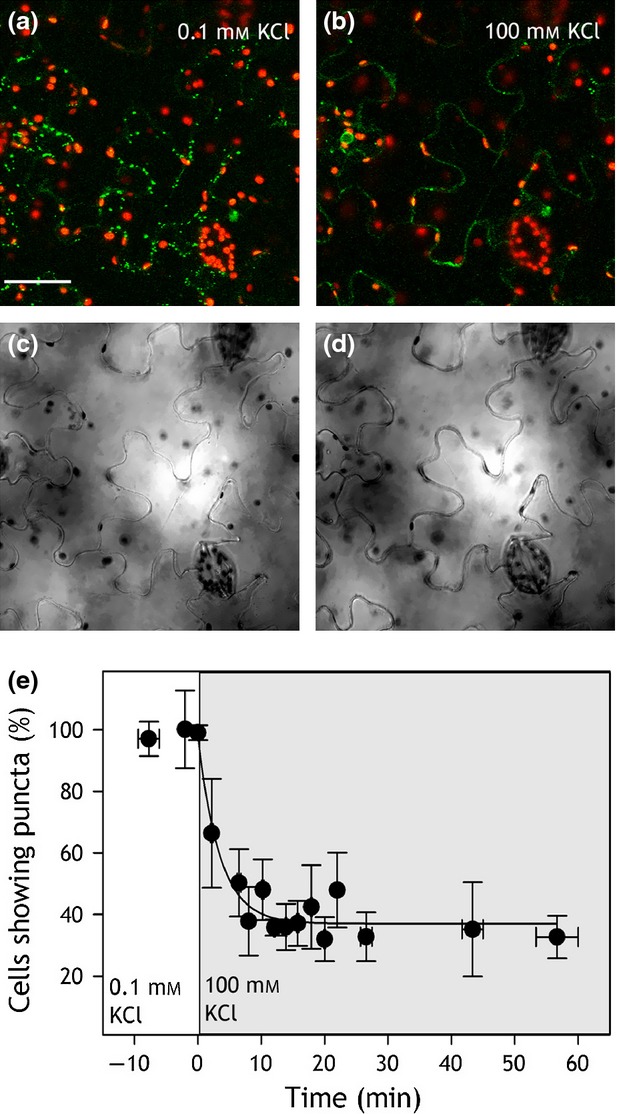
GORK-GFP puncta disperse rapidly on transfer to 100 mm KCl.(a–d) Confocal images (a, b) of GORK-GFP (green) and chloroplast fluorescence (red) expressed in tobacco epidermal cells with corresponding bright field (c, d) images collected in 0.1 mm KCl (a, c) and 30 min after transfer to 100 mm KCl (b, d) showing conversion from punctate to diffuse distribution of the channel. Scale bar, 50 µm.(e) The percentage of transformed tobacco cells displaying GORK-GFP puncta as a function of time on transfer from 0.1 to 100 mm KCl (grey). Data are means ±SE of *n* ≥ 5 independent experiments and were fitted by non-linear least-squares to an exponential decay function of time (solid line).

We analysed the time-course of puncta dispersal, expressing GORK in tobacco epidermis which showed the same reversible formation of puncta and simplified manipulations. Transformed leaf sections were infiltrated with 0.1 mm KCl and images collected before one half of the sections were re-infiltrated with 100 mm KCl. Samples were randomised and images were collected and analysed in blind assays following the second infiltration. The numbers of cells showing puntate distributions were assessed and then normalised to the measurements prior to the second infiltration. Figure5 summarises these data with images collected before (a,c) and after (b,d) infiltration with 100 mm KCl, and shows a rapid decrease in the percentage of cells showing puncta, the count dropping to approximately 40% (Figure5e) within 10 min of treatments. Exponential fittings yielded a halftime of 2.4 ± 0.5 min for the response. This analysis overestimates the halftime, as the experiments do not take into account the K^+^ diffusion time across the cell wall which can slow diffusion by a factor of 10^−2^ to 10^−4^ (Canny, 1990). Assuming a 10^−3^ -fold decrease, for K^+^ the effect implies a diffusion coefficient of 6.10^−9^ cm^2^ sec^−1^ and an increase in the root mean square time for bulk diffusion across the 2 μm of cell wall to 100–200 sec (Hille, 2001), roughly equivalent to the time for GORK-GFP puncta dispersal. These values contrast with the dispersion of KAT1-GFP puncta during endocytosis (Sutter *et al*., 2007), which displayed a halftime of 10–12 min and was complete only after 40–60 min following ABA treatments. The comparison supports our earlier observations indicating that the redistribution of GORK-GFP is not associated with endocytosis.

**Figure 6:**
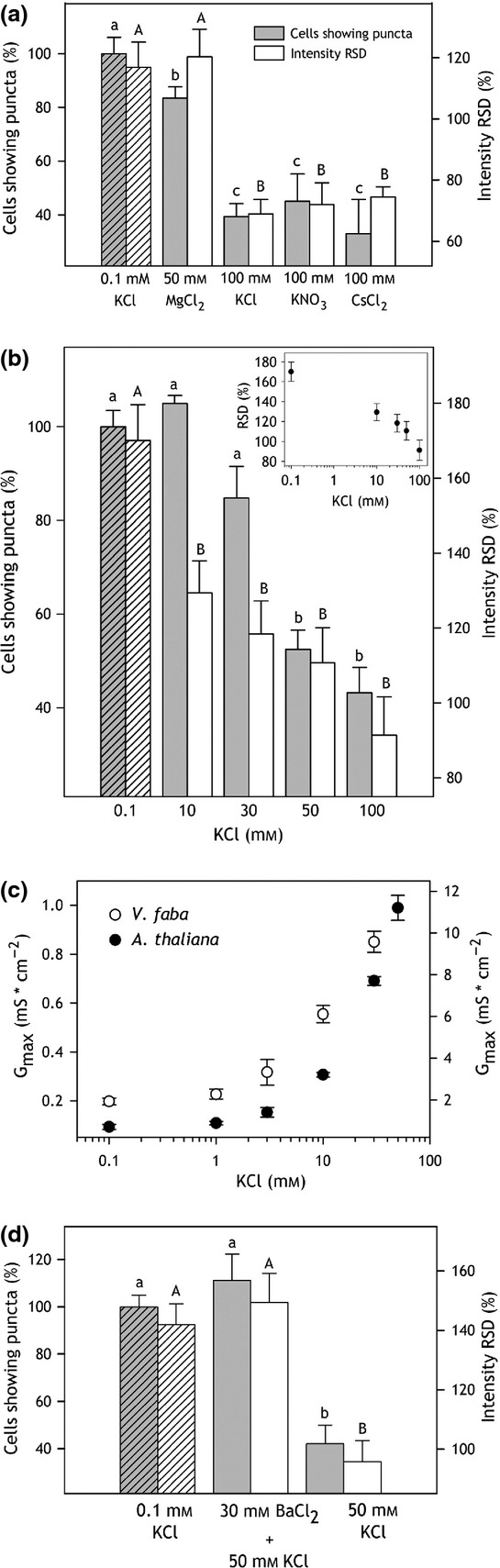
GORK-GFP dispersion is subject to K^+^, not Cl^−^, is equally sensitive to K^+^ substitution with Cs^+^, and is suppressed by pretreatment with the channel blocker Ba^2+^.(a) Quantification of GORK-GFP puncta in 0.1 mm KCl (diagonal shading) and after treatment with 100 mm KCl, 100 mm KNO_3_, 50 mm MgCl_2_ and 100 mm CsCl. Punctate distribution was analysed by two methods. The left ordinate and grey bars represent the percentage of cells showing puncta in any one image. The right ordinate and white bars represent the relative standard deviation (RSD) of intensities determined from a 1-pixel-wide line around the periphery of cells and normalized to the intensity means. Data are means ±SE of *n* > 9 independent experiments for each treatment.(b) Percentage of cells showing GORK-GFP puncta (left ordinate, grey bars) and the intensity SD (right ordinate, white bars) in 0.1 mm KCl (diagonal shading) and after treatment with 10, 30, 50 and 100 mm KCl. Data are means ±SE of *n* > 6 independent for each treatment. *Inset*: Intensity RSD as a function of [KCl] outside plotted on a logarithmic scale.(c) Maximum ensemble conductance of GORK in Arabidopsis guard cells (filled circles) and the homologous, outward-rectifying K^+^ current in *Vicia* guard cells (open circles) as a function of [K^+^] outside. Data are derived and replotted as means ± SE from prior publications [see (Blatt, 1988; Thiel and Blatt, 1991; Thiel *et al*., 1992; Blatt and Gradmann, 1997; Brearley *et al*., 1997; Blatt *et al*., 1999; Leyman *et al*., 1999; Sokolovski and Blatt, 2004; Chen *et al*., 2012; Wang *et al*., 2012)].(d) Percentage of cells showing GORK-GFP puncta (left ordinate, grey bars) and the normalized relative standard deviation in intensity (RSD; right ordinate, white bars) in 0.1 mm KCl (diagonal shading), after 30 min pretreatment with 30 mm of the K^+^ channel blocker BaCl_2_, and after addition of 50 mm KCl to the 30 mm BaCl_2_-treated samples. Data are means ±SE of *n* = 5 independent experiments. Significance at *P* < 0.05 was analysed by anova and pair-wise multiple comparison and are indicated in each case by lettering for the percentage of cells showing puncta (small letters) and RSD (capital letters).

The gating of GORK, and its homologs in *Vicia* and tobacco, responds over a similar time course to changes in alkali cation concentrations, including Cs^+^ (Blatt, 1988; Armstrong *et al*., 1995; Blatt and Gradmann, 1997; Blatt *et al*., 1999; Bauly *et al*., 2000). We examined whether the distribution GORK in puncta was sensitive to the K^+^ or to the Cl^−^ concentration, and whether it showed ionic sensitivities similar to those of gating. Experiments were carried out as before with pre-infiltrations of 0.1 mm KCl, but with either 100 mm KNO_3_ or 50 mm MgCl_2_ in the second infiltration. Leaf sections were examined for GORK-GFP distribution after 20 min. The results (Figure6a) showed a similar decrease in punctate frequency to 45 ± 10% in leaf sections treated with 100 mm KNO_3_ but not with 50 mm MgCl_2_. To avoid the ‘digitizing’ effect of counting cells, we also quantified GORK distribution by analysis of the relative standard deviations (RSD) in fluorescence intensity around the periphery of the cells. This approach yielded the same pattern in K^+^ dependence. As a further test, we also replaced the KCl solution with 100 mm CsCl. Again, these substitutions gave a punctate distribution of 33 ± 12% and a corresponding decrease in RSD, similar to that 100 mm KCl. We challenged leaf sections with different K^+^ concentrations for comparison with GORK channel gating. Leaf disks were pretreated as before, then split for blind image acquisition and the section fragments re-infiltrated with 0.1, 10, 30, 50 and 100 mm KCl. The results (Figure6b) showed that the percentage of cells with puncta decreased progressively with increasing KCl concentrations above 10 mm, although a statistical analysis belies the trend. This pattern is similar to that of the ensemble conductance maximum (*G*_max_) observed for the outward-rectifying K^+^ channels in both *Vicia* and Arabidopsis guard cells (Figure6c). Quantified by RSD, the effect of KCl concentration is evident across the entire concentration range. Finally, we tested whether the K^+^ channel blocker Ba^2+^ (Armstrong and Taylor, 1980; Roelfsema and Prins, 1997; Romano *et al*., 1998; Hamilton *et al*., 2000) might affect the distribution of GORK-GFP. In this case, leaf disks were pretreated with 0.1 mm KCl and split as before, one half of each disk then re-infiltrated either with 50 mm KCl or first with 30 mm BaCl_2_ and, after 30 min, with 30 mm BaCl_2_ plus 50 mm KCl. Figure6c shows that infiltrating with BaCl_2_ was sufficient to prevent the dispersal of the GORK puncta. These findings indicate a close association between GORK gating and the distribution of GORK-GFP in puncta and we return to the observations below.

## Discussion

Only recently has attention in channel regulation turned to the traffic of ion channel proteins and to changes in the population of ion channels active at the plasma membrane. Notable among the few studies in plants is the ABA-evoked endocytosis of the KAT1 K^+^ channel, its slower recycling to the plasma membrane (Sutter *et al*., 2007), and a role for the vesicle-trafficking protein SYP121 in recovery from programmed closure of guard cells (Eisenach *et al*., 2012). Until now, no information has been forthcoming relating to the traffic of GORK, the major outward-rectifying K^+^ channel that facilitates K^+^ efflux during stomatal closure in Arabidopsis, although changes in ensemble channel conductance and the voltage-dependence of gating are known for these and homologous outward-rectifying channels (Blatt, 1990, 1992; Thiel *et al*., 1992). We anticipated these effects on GORK current might result from channel traffic, but the studies outlined above contradict this expectation. Here we report that the GORK K^+^ channel, like KAT1, appears in discrete puncta at the guard cell periphery. These clusters form independently of the structures assembled by KAT1 (Meckel *et al*., 2004; Sutter *et al*., 2006, 2007) and are not affected by ABA, but form and disperse reversibly with external K^+^ concentration. Three additional lines of evidence associate GORK puncta and their dispersal with short-term changes in channel gating and conductance, rather than with channel traffic. (i) The mobility of GORK-GFP was unaffected by ABA, nor was it altered between the punctate and dispersed forms of the channel. (ii) Two-phase partitioning experiments yielded no substantial difference in membrane distribution between punctate and dispersed forms of the channel, unlike KAT1 (Sutter *et al*., 2007; Eisenach *et al*., 2012). (iii) Dispersal of the GORK puncta was rapid and demonstrably sensitive to extracellular cations, parallelling gating and block in these channels. We suggest that conformational changes of the channel and its gate with K^+^ are connected structurally to the clustering of GORK within the plane of the plasma membrane. Such clustering may signal changes in the external ionic environment, possibly by exploiting the changing capacity of GORK, when in puncta, to interact with other cytoplasmic or plasma membrane proteins.

### GORK puncta are distinct from the trafficking platforms of the KAT1 channel

Clustering of the GORK K^+^ channels within puncta at the surface of tobacco epidermal cells and Arabidopsis guard cells complements studies of the other major K^+^ channel subgroup, represented by KAT1 (Meckel *et al*., 2004; Sutter *et al*., 2006, 2007; Homann *et al*., 2007), and supports electrophysiological evidence that many functional K^+^ channels in plants are distributed non-homogenously over the plasma membrane surface (Tester, 1990; Hille, 2001). The puncta formed by GORK are similar in size to those of KAT1, but are physically distinct as evident from co-localisation studies (Figures1, S3 and S4). GORK puncta also differ from those of KAT1 in their mobility and sensitivity to external stimuli. Whereas the puncta of KAT1 are lost in ABA (Sutter *et al*., 2007) and reform in a manner sensitive to the vesicle-trafficking protein SYP121 (Eisenach *et al*., 2012), we found GORK to be insensitive to ABA and SYP121 but dependent on the extracellular K^+^ concentration (Figures2, 5 and 6). These findings alone distinguish the structural characteristics of the two K^+^ channels.

Physical clustering of channel and other proteins is well known also in animal cells (Lai and Jan, 2006; O'Connell *et al*., 2010; Zilly *et al*., 2011; Pristera *et al*., 2012) and, in neurons, often demarcates platforms for vesicle fusion and channel traffic (Lai and Jan, 2006; Deutsch *et al*., 2012). Much the same conclusion was reached for the puncta formed of the KAT1 channel when expressed in tobacco (Sutter *et al*., 2006, 2007): Our analysis of GORK-GFP recovery after photobleaching suggested that roughly 10% may be mobile within the cell (Figure3), possibly through vesicle traffic. However, two-phase partitioning failed to uncover a substantial change in GORK distribution between plasma- and endomembranes fractions, even following transfer to 100 mm KCl to evoke a dispersal of the puncta. GORK puncta also dissociated reversibly in the *syp121* mutant that is defective in KAT1 trafficking to the plasma membrane (Eisenach *et al*., 2012). We cannot discount a role for vesicle traffic in the mobility of GORK, but these results indicate that any such contribution to GORK clustering and dispersal is small and of a magnitude that might only be resolved by fluorescence analysis *in vivo* over extended time periods (Luu *et al*., 2012).

We stress that plant ion channels, including GORK, normally express at levels that are too low for detection by confocal microscopy. We used GORK-GFP constructs driven by the Ubiquitin-10 promoter, *pUBQ10*, that drives expression at lower levels that the 35S CaMV promoter (Grefen *et al*., 2010b), and we confirmed channel function of GORK-GFP. We cannot rule out that *pUBQ10*-driven expression misrepresents GORK distribution. Nonetheless, GORK-GFP generally was not observed to accumulate in the endoplasmic reticulum, and GORK puncta dissociated reversibly *in vivo*. These findings support the idea that clustering of the K^+^ channels is physiologically relevant. Clustering of membrane proteins in lipid rafts is common and often accompanied by restricted lateral diffusion (Bhat *et al*., 2005; Lai and Jan, 2006; Mongrand *et al*., 2010). Such rafts have been implicated in signal transduction, polarised secretion, membrane traffic and pathogen entry. Membrane rafts are generally acknowledged to form below the resolution limit of the light microscope and, thus, are 5- to 10-fold smaller than the GORK-GFP clusters we observed. However, such small-scale rafts may aggregate in response to external stimuli (Bhat *et al*., 2005; Mongrand *et al*., 2010), potentially underpinning the larger clusters observed with the GORK K^+^ channel. Our FRAP analysis (Figure3) showed that cluster dispersion was not associated with a significant increase in mobility or altered macroscopic diffusion. Thus, it may be that GORK remains anchored with other proteins in raft-like microdomains, even in high external K^+^.

### A physiological role for GORK clustering associated with K^+^-dependent gating?

The most striking aspects of the physical organisation of GORK are its parallels to the K^+^-sensitivity of gating and ensemble conductance. The activities of the Kv-like channels GORK and SKOR in Arabidopsis, as well as outward-rectifying K^+^ channels in other plants, show a characteristic dependence on external alkali cation concentration as well as voltage (Blatt, 1988; Armstrong *et al*., 1995; Blatt and Gradmann, 1997; Blatt *et al*., 1999; Ache *et al*., 2000; Bauly *et al*., 2000; Johansson *et al*., 2006). These characteristics include a displacement in channel conductance in parallel with the equlibrium voltage for K^+^ (see Figure S1), an increase in the ensemble conductance maximum, and the ability of Cs^+^ to substitute for K^+^ outside. Channel gating responds in a near-instantaneous manner to K^+^ within the timescale of bulk solution changes, and changes in ensemble conductance are only marginally slower (Blatt, 1988; Blatt and Gradmann, 1997). Johansson *et al*. (2006) have shown that external cations affect the K^+^ channel through interactions of the pore loop and pore-lining α-helices that, in turn, are likely to influence the conformational states of the outer ring of polypeptides formed by the voltage-sensor domains of the channels (Dreyer and Blatt, 2009): in effect, occupancy of the inner vestibule of the pore at high K^+^ concentrations appears to favour a temporally-stable, ‘locked-closed’ state. We speculate that these conformations may affect the local packing of the K^+^ channel proteins within the plasma membrane, which becomes evident at a macroscopic level as cluster dissolution. Barium suppression of the GORK puncta dispersal (Figure6), and the parallels in timecourse for cluster dissolution and gating in 100 mm K^+^, makes this hypothesis especially compelling. Barium enters the pores of many K^+^ channels (Vergani *et al*., 1997; Hamilton *et al*., 2000; Jiang and MacKinnon, 2000; Hille, 2001), including GORK (Roelfsema and Prins, 1997; Ache *et al*., 2000), and is thought to block by occluding the selectivity filter (Jiang and MacKinnon, 2000; Rowley and Roux, 2013). It follows that, with Ba^2+^ present, the channel is likely to become trapped in the ‘locked-closed’ state with K^+^ in the inner vestibule of the channel, isolated from the pool of K^+^ in the cytosol and, of course, from changes in K^+^ concentration outside. Thus, the effect on GORK cluster dispersal implies a close connection to channel conformation in gating and to channel conductance.

What role might reversible clustering of the K^+^ channels serve? In addition to the voltage-dependence of the channel gate, at least two physiological phenomena may be connected to this behaviour. First, we suggest that clustering may serve to control the pool of GORK channels active in the plasma membrane by sequestering channels, possibly in a non-activatable state. Such behaviour might be seen to withdraw channels from the active pool at the membrane much as an iceberg constrains a volume of water within a crystalline array, preventing its movement and ability to act as a general solvent. For the channels at low external K^+^ concentrations, the effect of such an ‘iceberg’ model might be seen to confer a synergy to the voltage-dependence of the channels as the K^+^ concentration decreases while reducing the maximum K^+^ flux capacity of the channel population (Blatt, 1988, 1990, 1992; Thiel *et al*., 1992; Armstrong *et al*., 1995; Blatt *et al*., 1999; Bauly *et al*., 2000) (see also Figure6). Similar concepts have been proposed to explain the clustering of mammalian K^+^ channels (Misonou and Trimmer, 2004), although correlating channel clustering and ensemble conductance has proven difficult (O'Connell *et al*., 2010).

We can propose a second role for GORK clustering that builds on this iceberg analogy. K^+^ flux across the plasma membrane is known to be tightly linked to membrane surface area and volume control (Zonia and Munnik, 2007). Work from this laboratory has uncovered the wide-spread interaction between subsets of vesicle-trafficking (SNARE) proteins and K^+^ channels that have profound effects on channel gating and net K^+^ uptake (Honsbein *et al*., 2009, 2011; Grefen *et al*., 2010a). These interactions are implicated in priming the SNAREs to catalyse membrane fusion (Karnik *et al*., 2013). It is plausible, therefore, that the cell might exploit the K^+^-sensitivity of GORK clustering to regulate vesicle traffic of other membrane components, adjusting the rate of cell surface expansion with K^+^ uptake and cell volume. For example, K^+^ accumulation is enhanced in guard cells at high external K^+^, which also promotes the dissolution of GORK clusters. We can imagine that ‘freeing’ the channels from within the clusters facilitates SNARE and other interactions to accelerate vesicle traffic for the increase in cell volume.

In conclusion, we have uncovered a new and unusual process that may help to understand how the K^+^ channels of Arabidopsis guard cells, and other plant cells, sense and respond to external K^+^ concentration. To date, the phenomenon of K^+^-dependent channel clustering in plants appears unique to the outward-rectifying K^+^ channel GORK, but we anticipate that similar characteristics will be forthcoming for other outward-rectifying K^+^ channels in plants. For GORK, the properties of channel clustering shows many hallmarks of K^+^-dependent gating and conductance, implicating a close connection that is now open to testing, for example through a combination of protein domain exchange with a non-clustering channel such as AKT1 (Honsbein *et al*., 2009). We suspect that the organisation of GORK and other channels in this manner is likely to contribute both as a mechanism regulating the channels and as a platform for sensing and responding to environmental cues.

## Material and Methods

### Molecular cloning

The GORK coding sequence was amplified from rosette-leaf cDNA using the oligonucleotide primers 5′-attB1 TA ATG GGA CGT CTC CGG-3′ and 5′-attB2 G TGT TTG ATC AGT AGT ATC ACT G-3′, recombined into Gateway™ entry vector pDONR207 with BP clonase II (Life Technologies, http://www.lifetechnologies.com), and transformed into the *E. coli* strain *CopyCutter EPI400* (Cambio, http://www.cambio.co.uk) (Grefen *et al*., 2009). Clones were verified by sequencing (GATC, http://www.gatc-biotech.com/) and recombined into expression vectors via LR-reaction (Life Technologies). The *pUBQ10* vector system was used to generate GORK constructs C-terminally tagged with GFP and RFP (Grefen *et al*., 2010b). Constructs were transformed into *Agrobacterium tumefaciens* (strain GV3101) and were verified by rescue in *E. coli* and restriction digest analysis. A single *A. tumefaciens* clone was used for transient transformation of tobacco and stable transformation of Arabidopsis.

The coding sequence for GORK-GFP was amplified from the *pUBQ10 -DEST* expression construct for re-cloning into the oocyte expression vector *pGT-DEST* (Grefen *et al*., 2010b) using the oligonucleotide primers 5′-A GTT AAC ATG GGA CGT CTC CGG AGA C-3′ and 5′-C AGG CCT TTA TAA CTT GTA CAG CTC GTC CAT GC-3′. These primers incorporated 5′ *HpaI* and *StuI* overhangs, respectively. Blunt-end restriction of the oocyte expression vector *pGT-DEST* with *MscI* and of the PCR product with *HpaI* and *StuI* was followed by ligation using T4 DNA Ligase (Promega, http://www.promega.com/) before verification by sequencing.

### Electrophysiology

GORK-GFP was expressed in *Xenopus* oocytes using *pGT GORK-GFP* plasmid DNA (Vergani *et al*., 1998; Grefen *et al*., 2010a), and currents were recorded by two-electrode voltage clamp as described previously (Johansson and Blatt, 2006). Measurements were carried out using microelectrodes filled with 3 m K^+^-acetate and with oocytes bathed in 10 mm HEPES-NaOH, pH 7.2, with 1.8 mm MgCl_2_, 1.8 mm CaCl_2_ and additions of 10 mm, 30 mm or 96 mm KCl balanced by NaCl to give a total salt concentration of 100 mm.

### Plant growth, transformation and microscopy

*Arabidopsis thaliana* Col0, *syp121* and *gork* [SALK_082258C (Alonso *et al*., 2003)] mutant plants were grown and transformed by floral dip (Clough and Bent, 1998) and T_1_ transformants were selected for BASTA® resistance (1:1000 dilution; Bayer Cropscience, http://www.cropscience.bayer.com). Seeds of the T_2_, T_3_ and T_4_ generations were selected in the same manner. Leaves of *Nicotiana tabacum* (tobacco) were transformed by infiltration with *Agrobacterium* carrying the GORK-GFP construct (Geelen *et al*., 2002), and were analysed 72–120 h after transformation.

GORK-GFP fluorescence was resolved on a LSM510-META confocal microscope (Zeiss, http://www.zeiss.com) using the 488-nm line of an Argon laser and fluorescence was collected as reflectance from a 545-nm dichroic mirror after passage through a 505–530 nm interference filter. Chloroplast fluorescence was collected concurrently after passage through the 545-nm dichroic mirror and a long pass 560 nm emission filter. Fluorescence recovery after photobleaching (FRAP) was carried out after fluorophore bleaching with the 488 laser using a pixel time of 1.6 sec (2.4 mJ exposure energy). Bleaching, detection and analysis settings were standardised between experiments for ROI dimensions, laser power, magnification and detection settings and fluorescence was corrected for decay during acquisition.

Imaging was carried out using young rosette leaves of Arabidopsis mounted in a custom-build perfusion chamber that enabled continuous perfusion and solution changes during experiments. Buffers included 0.003% Silwet L77 (Lehle Seeds, http://www.lehleseeds.com) to ensure cuticle penetration. For tobacco, imaging was carried out on 1-cm diameter leaf discs after vacuum infiltration with treatment buffers before remounting. All buffers were based on 10 mm NaMES, pH6.1. The salts KCl, KNO_3_, CsCl_2_, MgCl_2_ and BaCl_2_ were added as indicated and osmotic strength was adjusted using mannitol. Abscisic acid (ABA) was prepared as before (Blatt and Armstrong, 1993) and diluted in buffer to 40 μm for use. For ABA treatments, leaves were infiltrated and incubated in 5 mm Ca^2+^-MES, pH 6.1, 10 mm KCl for 2 h under 100 μmol m^−2^ sec^−1^ white light. Images were collected, the leaves reinfiltrated using the same buffer with the addition of ABA, and the leaves re-imaged after 30 and 60 min. Tobacco disc analysis was carried out in blind assays. GORK puncta were quantified both as the percentage of cells showing puncta and by relative standard deviation (RSD) after normalizing to the intensity means.

### Protein biochemistry

GORK distribution between plasma membrane and endomembrane fractions was determined by aqueous two-phase partitioning. Arabidopsis leaf tissues were infiltrated in either 100 mm KCl or 0.1 mm KCl buffers and incubated for 2 h. All subsequent steps were carried out at 4°C as described before (Honsbein *et al*., 2009). Proteins were separated by electrophoresis and transferred onto PVDF membranes (Honsbein *et al*., 2009; Karnik *et al*., 2013) and were probed overnight with primary antibody, either αGORK [1:100]; αAHA [1:5000, (Villalba *et al*., 1991)], αSec61 [1:3000, (Yuasa *et al*., 2005)], or αGFP [1:200; Abcam, http://www.abcam.co.uk]. Horseradish peroxidase-coupled, α-rabbit IgG secondary antibody (1:100 000) was used for detection with WestFemto SuperSignal (Pierce; Thermo Scientific, http://www.thermofischer.com). In some experiments, membranes were re-probed after stripping by incubation in 100 mm β-Mercaptoethanol, 2% SDS, 62.5 mm Tris-HCl, pH 6.7 at 70°C for 45 min.

### Data analysis

Statistical analyses were carried out using sigmaplot 11 (Systat Software, Inc., http://www.sigmaplot.com). Non-linear least-squares fittings used a Marquardt–Levenberg algorithm (Marquardt, 1963). Significance was tested using Student's *t*-test and analysis of variance (anova). Otherwise, data are reported as means ± SE of *n* observations.

### Chemicals and media

All chemicals were from Sigma (http://www.sigma-aldrich.com) unless otherwise noted.

## Supporting Information

Figure S1 The GFP-tagged GORK construct encodes a functional K^+^ channel.

Figure S2 GORK current is blocked by the K^+^ channel antagonist tetraethylammonium chloride (TEA^+^).

Figure S3 The K^+^ channels GORK and KAT1 assemble in physically-distinct puncta.

Figure S4 KAT1-GFP and GORK-RFP show intermediate degree of overlap, suggesting that the two channel proteins do not coreside in the same punctate structures at the plasma membrane.

Figure S5 GORK-GFP clusters appear in strands of plasma membrane on cell plasmolysis.

Figure S6 GORK-GFP clusters are unaffected by ABA.

Figure S7 Relative standard distribution (RSD) of GORK-GFP at the guard cell periphery is unaffected by ABA.

Figure S8 GORK peptide antigen design.
