# Supplementary figures and images for: Clustering of the K+ channel GORK of Arabidopsis parallels its gating by extracellular K+
Source: Plant J. 2014 Apr 2;78(2):203–14. doi: 10.1111/tpj.12471 (PMC4309415; doi:10.1111/tpj.12471)

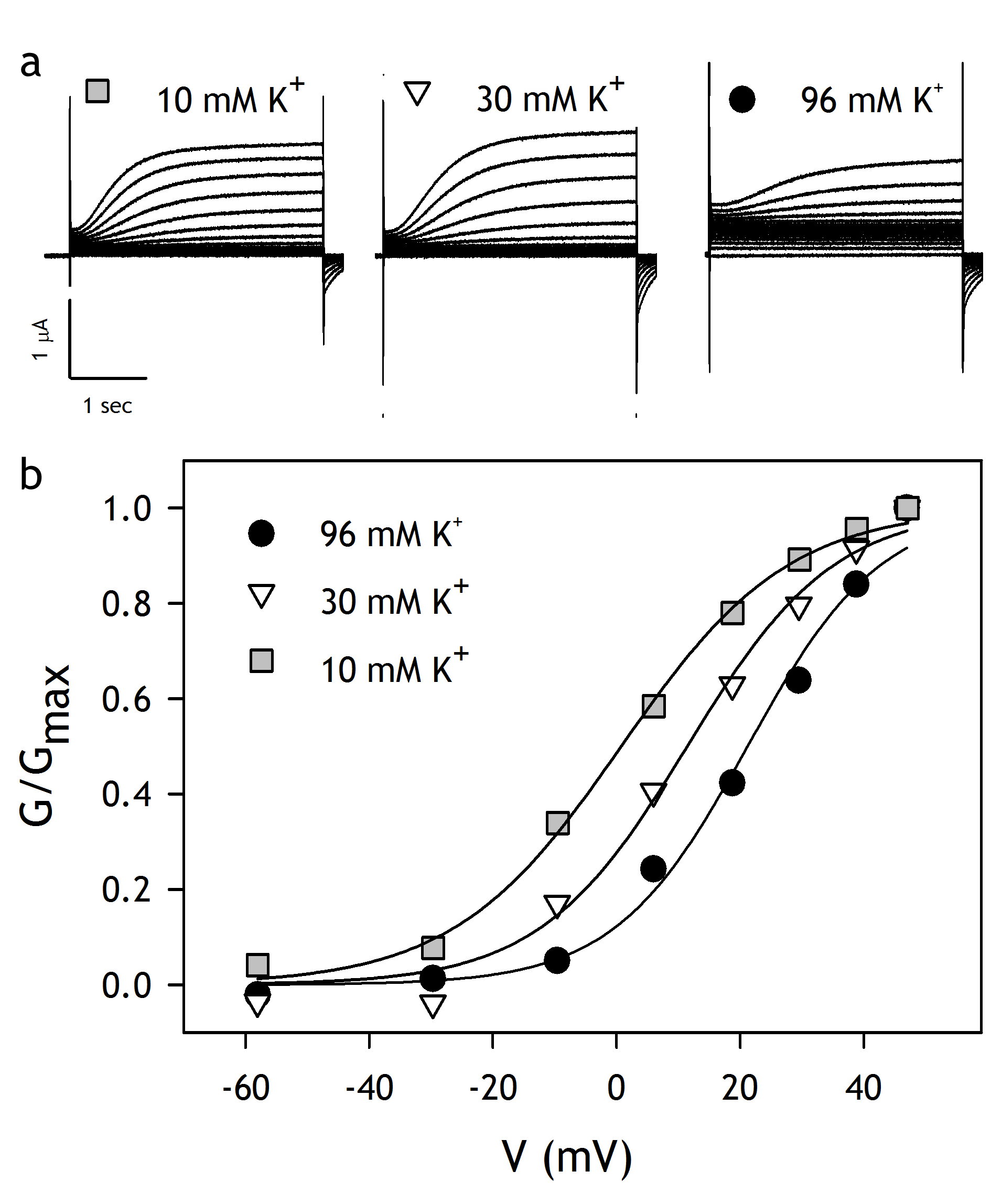

Supplement: Figure S1 — The GFP-tagged GORK construct encodes a functional K+ channel. [file tpj0078-0203-sd1.tif]

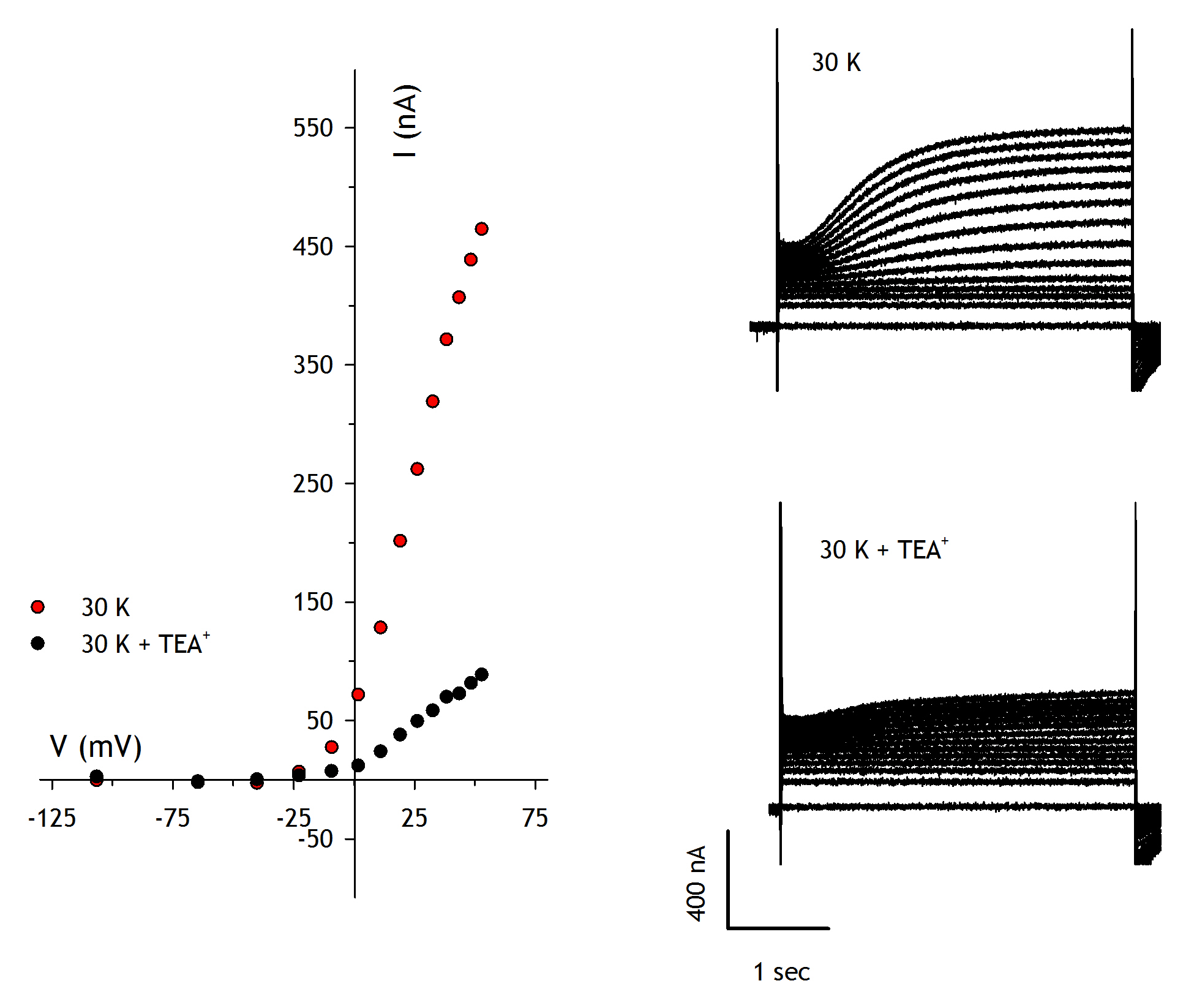

Supplement: Figure S2 — GORK current is blocked by the K+ channel antagonist tetraethylammonium chloride (TEA+). [file tpj0078-0203-sd2.tif]

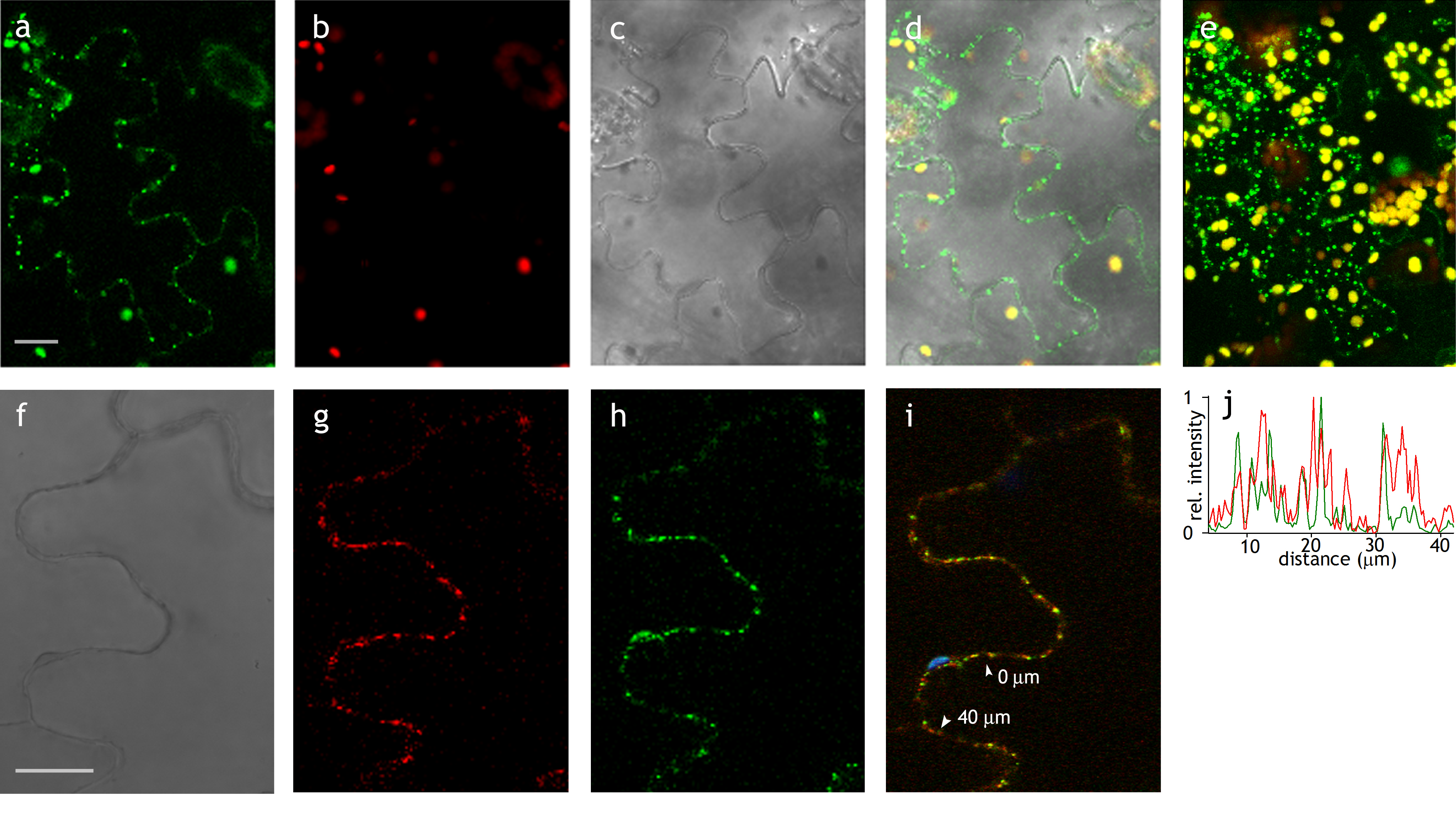

Supplement: Figure S3 — The K+ channels GORK and KAT1 assemble in physically-distinct puncta. [file tpj0078-0203-sd3.tif]

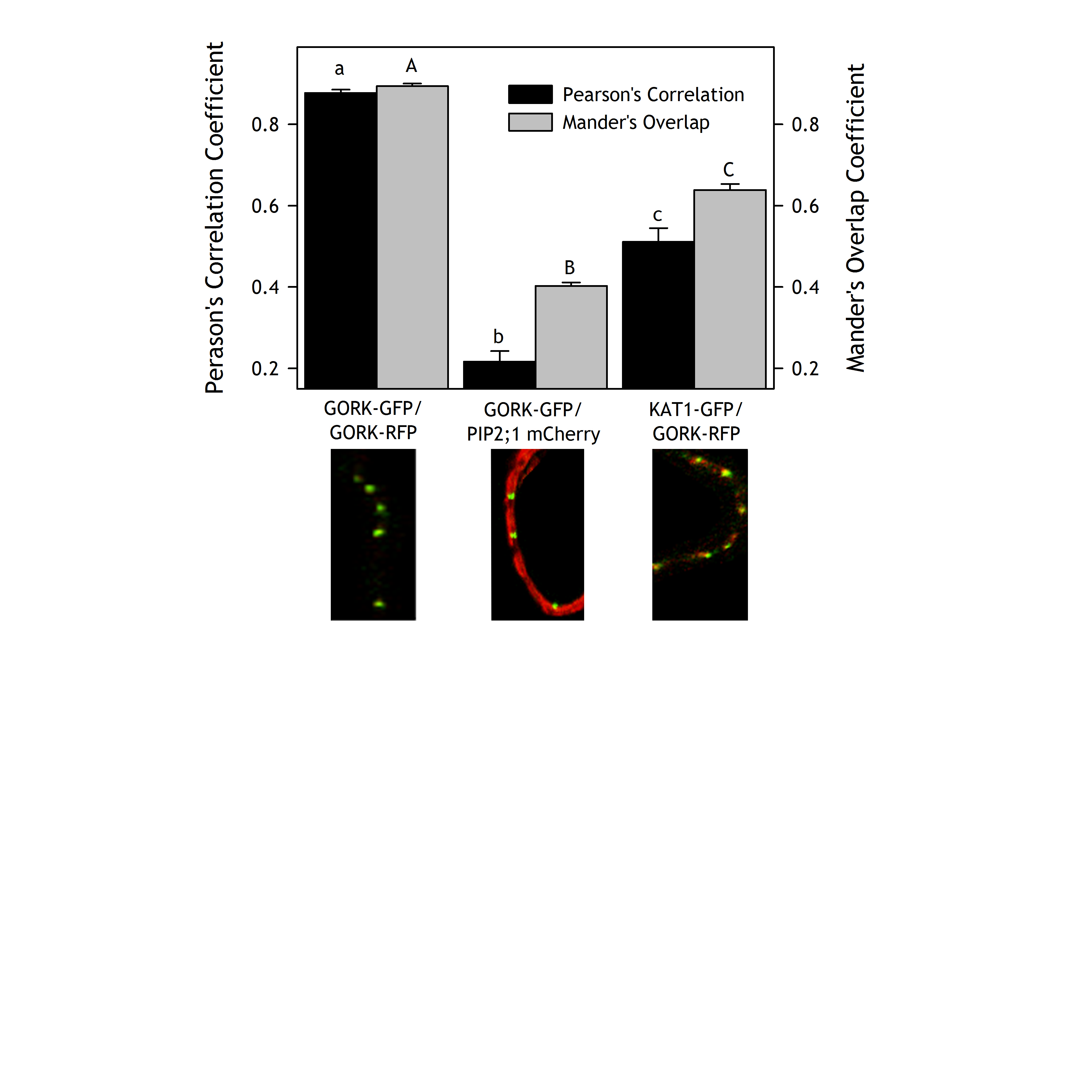

Supplement: Figure S4 — KAT1-GFP and GORK-RFP show intermediate degree of overlap, suggesting that the two channel proteins do not coreside in the same punctate structures at the plasma membrane. [file tpj0078-0203-sd4.tif]

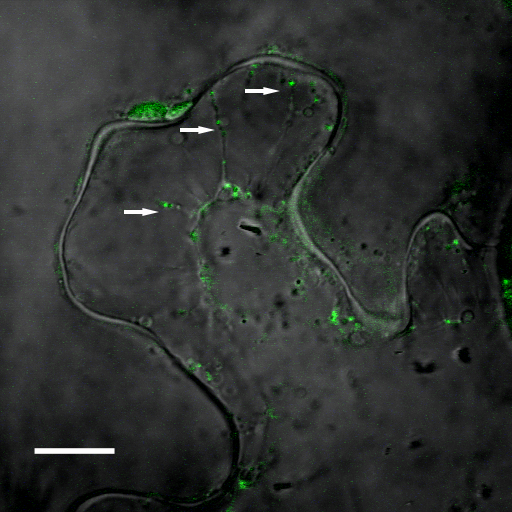

Supplement: Figure S5 — GORK-GFP clusters appear in strands of plasma membrane on cell plasmolysis. [file tpj0078-0203-sd5.tif]

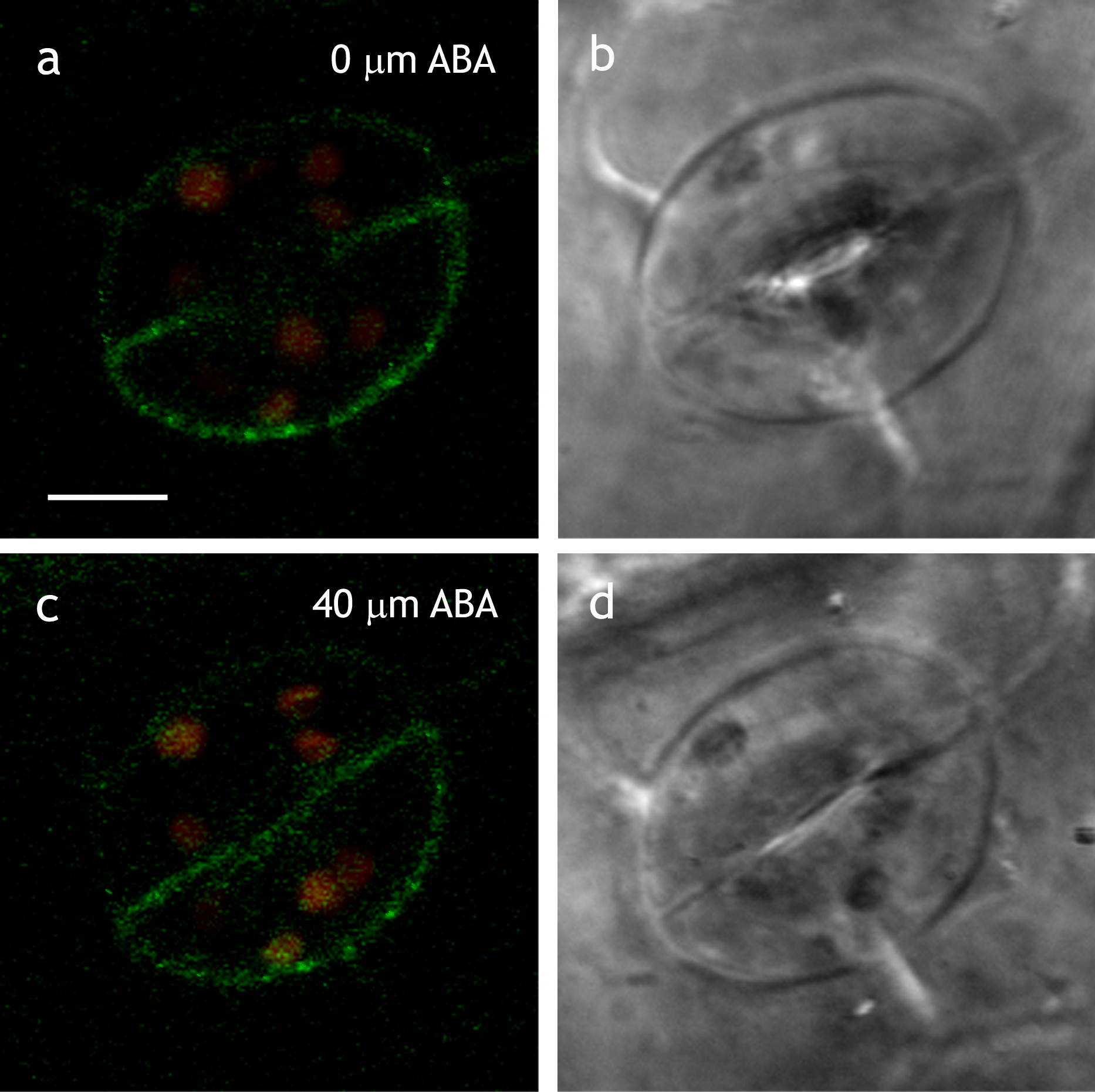

Supplement: Figure S6 — GORK-GFP clusters are unaffected by ABA. [file tpj0078-0203-sd6.tif]

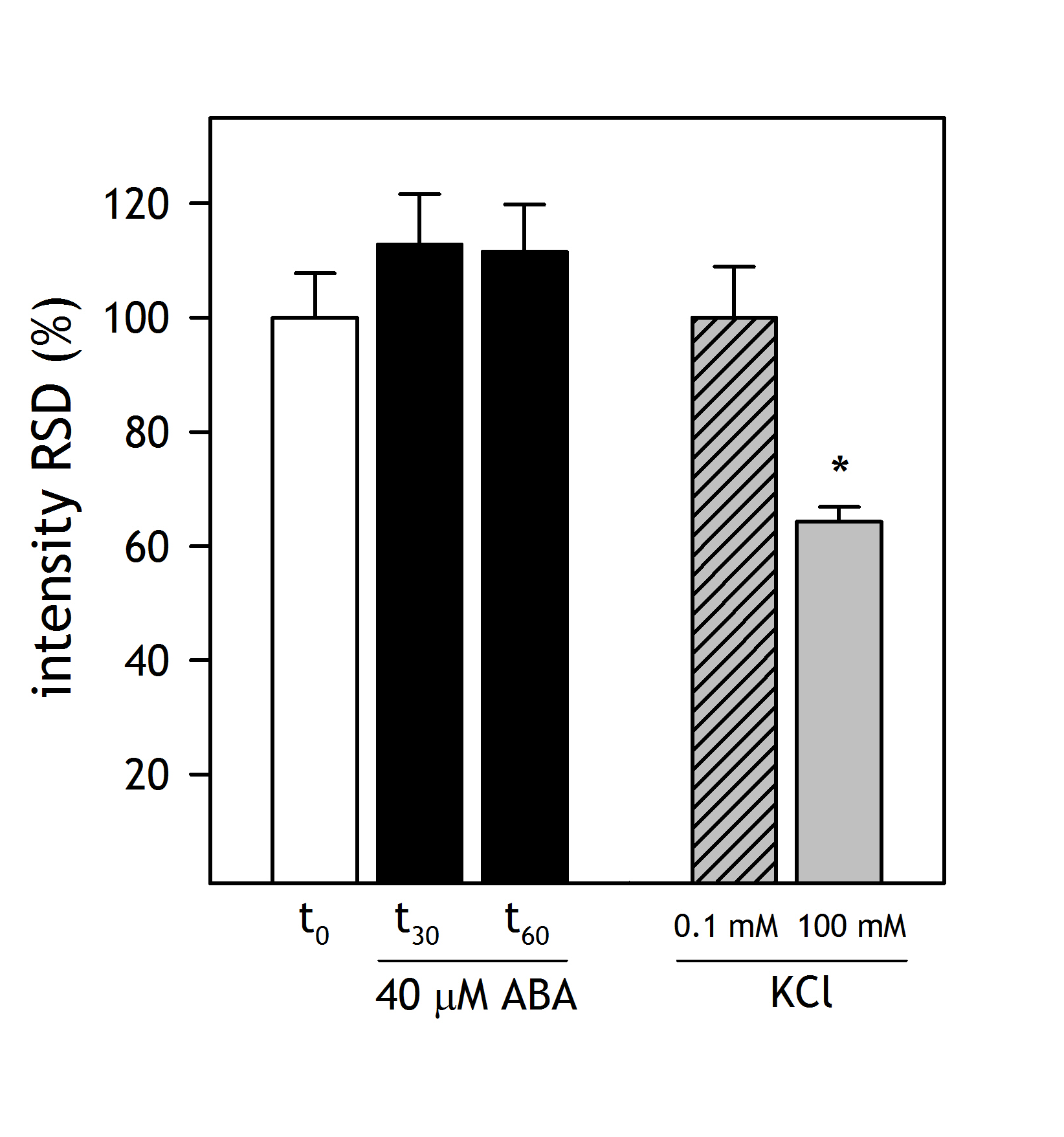

Supplement: Figure S7 — Relative standard distribution (RSD) of GORK-GFP at the guard cell periphery is unaffected by ABA. [file tpj0078-0203-sd7.tif]

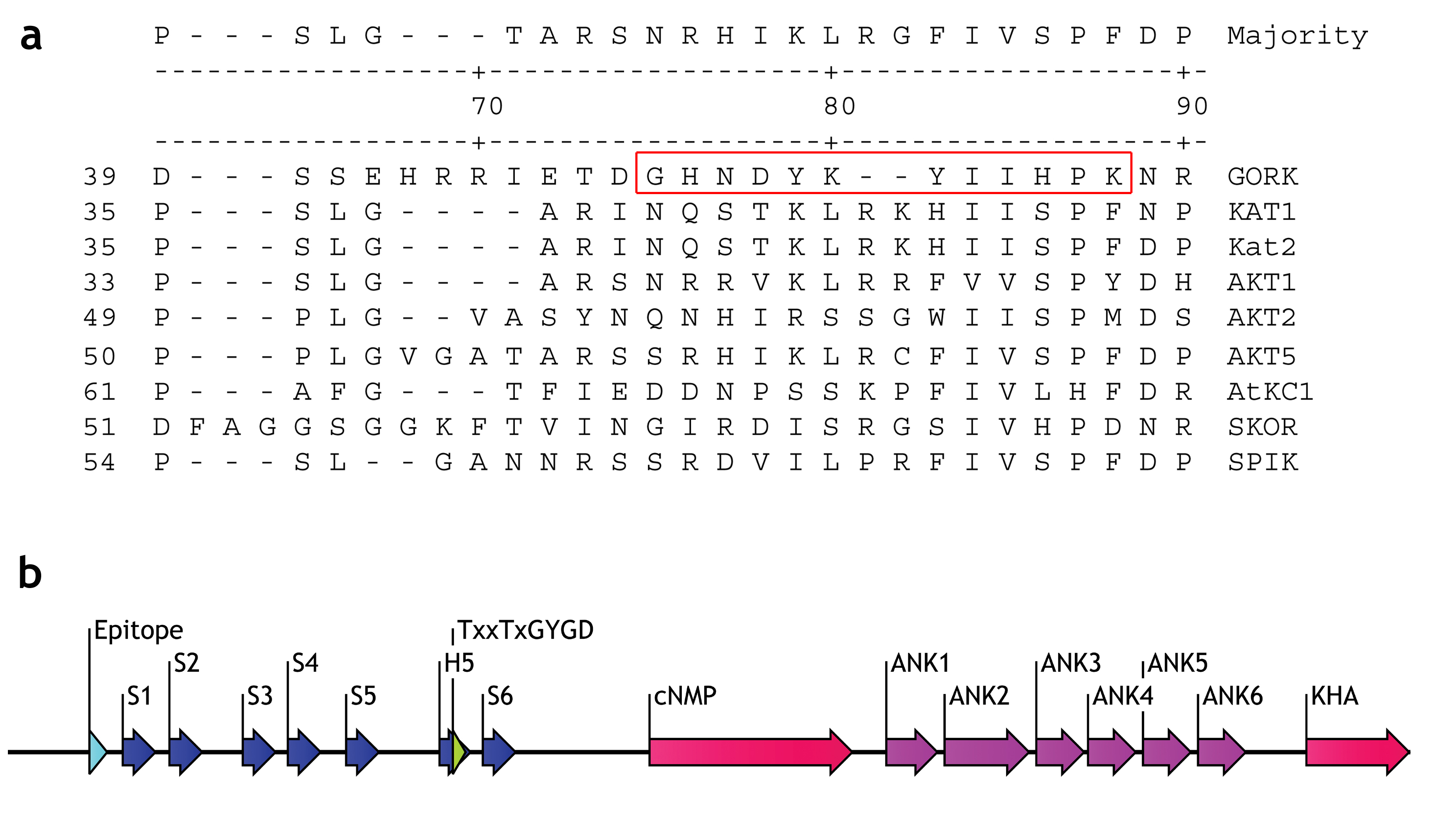

Supplement: Figure S8 — GORK peptide antigen design. [file tpj0078-0203-sd8.tif]
