# Supplementary material for: Clustering of the K+ channel GORK of Arabidopsis parallels its gating by extracellular K+
Source: Plant J. 2014 Apr 2;78(2):203–14. doi: 10.1111/tpj.12471 (PMC4309415; doi:10.1111/tpj.12471)
Supplement: Supplementary file 9 [file tpj0078-0203-sd9.doc]

**Clustering of the K^+^ channel GORK of Arabidopsis**

**parallels its gating by extracellular K^+^**

Cornelia Eisenach, Maria Papanatsiou, Ellin-Kristina Hillert, Michael R. Blatt

Laboratory of Plant Physiology and Biophysics, Institute of Molecular, Cell and Systems Biology, Bower Building, University of Glasgow, G12 8QQ, UK

Fig. S1. The GFP-tagged GORK construct encodes a functional K^+^ channel.

a) Current traces recorded under voltage clamp from oocytes expressing GORK-GFP. Data recorded from one oocyte in 10 mM (grey square), 30 mM (open triangle), 96 mM K^+^ (closed circle). Voltage was stepped from a holding voltage of -100 mV to voltages from -60 to +80 mV in -20 mV increments before returning to -100 mV.

b) Conductance-voltage curves, normalised to the conductance at +50 mV, in 10 mM (grey square), 30 mM (open triangle) or 96 mM K^+^ (closed circle). Data are fitted to the Boltzmann equation

[1]

where δ is the gating charge, V_1/2_ is the half-maximal activation voltage and F, R and T have their usual meaning. Fittings (solid lines) were obtained allowing only V_1/2_ to vary between curves, yielding *V_1/2_* values of +21±1 mV (96 mM K^+^), +11.4±1 mV (30 mM K^+^), -8±1 mV (10 mM K^+^).

Fig. S2. GORK current is blocked by the K^+^ channel antagonist tetraethylammonium chloride (TEA^+^).

Currents were recorded under voltage clamp in 30 mM KCl and 30 mM KCl with 10 mM TEA^+^. The corresponding current traces are shown on the right. Data from one oocyte. Similar results were obtained in each of 4 independent experiments.

Fig, S3. The K^+^ channels GORK and KAT1 assemble in physically-distinct puncta.

a-e) Confocal images of GORK-GFP (green, a) expressed in tobacco epidermal cells with corresponding chloroplast fluorescence (red, b), bright field (c) and overlay image (d). A 3D projection (e) of the GORK-GFP and chloroplast fluorescence signals, reconstructed from a z-stack collected at 3 µm intervals. Scale bar, 20µm.

f-j) Confocal images collected from tobacco epidermis expressing expressing GORK-RFP (red, g) and KAT1-GFP (green, h) with the brightfield image (f). Scale bar, 20µm. The corresponding overlay image (i) of RFP and GFP fluorescence was analysed for co-localisation by comparing intensity over position (j) around the cell periphery indicated by arrowheads in (i) and shows that KAT1-GFP and GORK-RFP yield distinct peaks which do not uniformly coincide.

Fig. S4. KAT1-GFP and GORK-RFP show intermediate degree of overlap, suggesting that the two channel proteins do not coreside in the same punctate structures at the plasma membrane. Intensity correlation analysis of RFP and GFP fluorescent signals at the cell periphery for three different co-expression experiments. *Left* to *right* are analyses for co-expression of GORK-GFP with GORK-RFP, of GORK-GFP with PIP2;1-mCherry, and of KAT1-GFP with GORK-RFP. Bars are Pearson’s correlation coefficient (black bars) and Mander’s overlap coefficient (grey bars). Representative images are shown below each coefficient pair. Data are from >6 independent experiments in each case and are given as means ±SE. Significance at P<0.05 is indicated by the lettering above each bar.

Fig. S5. GORK-GFP clusters appear in strands of plasma membrane on cell plasmolysis.

GORK-GFP fluorescence is shown overlaid on the brightfield image of a tobacco epidermal cell following plasmolysis with 0.85 M NaCl. Hectian strands of the plasma membrane are visible (*arrows*) with punctate GFP fluorescence. Scale, 10 μm.

Fig. S6. GORK-GFP clusters are unaffected by ABA. Representative images for one guard cell pair showing GORK-GFP fluorescence (a,c) and brightfield images (b,d) in 5 mM Ca^2+^-MES, pH 6.1 with 10 mM KCl before (a,b) and 60 min after (c,d) transfer to 40 μM abscisic acid (ABA). Scale bar, 5µm. Images in (a) and (c) are medial 3D projections of GORK-GFP (green) fluorescence from z-stacks taken at intervals of 0.7 µm. See also Fig. S7.

Fig. S7. Relative standard distribution (RSD) of GORK-GFP intensities at the guard cell periphery is unaffected by ABA. RSD analysis of 5 independent experiments with Arabidopsis leaves infiltrated with 5 mM Ca^2+^-MES, pH 6.1 with 10 mM KCl before (t_o_) , and 30 (t_30_) and 60 min (t_60_) after re-infiltration with the same buffer including 40 μM abscisic acid (ABA). Data for parallel experiments with 0.1 and 100 mM KCl (see Fig. 6) for comparison. Asterisk indicates statistical difference for 100 mM KCl vs 0.1 mM KCl at P <0.05.

Fig. S8. GORK peptide antigen design.

(A) Near N-terminal amino acid alignment of GORK and the other Arabidopsis Kv-like channel family members with the unique the epitope (resiudes 50-61, GHNDYKYIIHPK) used for antibody production indicated (*red box*).

(B) GORK protein model showing the epitope and structural features, including the six transmembrane domains (S1 – S6), the pore loop (H5) and K^+^ channel motif (TxxTcGYGD), a cyclic nucleotide binding domain (cNMP), ankyrin binding domains (ANK1 – ANK6), and a KHA domain. Both, N and C-terminus are cytoplasmic.
